# Dietary administration of β-1,3/1,6-glucan and *Lactobacillus plantarum* improves innate immune response and increases the number of intestine immune cells in roach (*Rutilus rutilus*)

**DOI:** 10.1186/s12917-020-02432-1

**Published:** 2020-06-26

**Authors:** Barbara Kazuń, Joanna Małaczewska, Krzysztof Kazuń, Rafał Kamiński, Dobrochna Adamek-Urbańska, Joanna Żylińska-Urban

**Affiliations:** 1 Department of Fish Pathology and Immunology, Stanisław Sakowicz Inland Fisheries Institute, Olsztyn, Poland; 2 Department of Microbiology and Clinical Immunology, Faculty of Veterinary Medicine, University of Warmia and Mazury, Olsztyn, Poland; 3 Pond Fishery Department, Stanisław Sakowicz Inland Fisheries Institute, Olsztyn, Poland; 4 Department of Ichthyology and Biotechnology in Aquaculture, Institute of Animal Sciences, Warsaw University of Life Sciences, Warsaw, Poland; 5 Department of Microbial Biochemistry, Institute of Biochemistry and Biophysics of the Polish Academy of Sciences, Warsaw, Poland; 6 Department of Technology and Biotechnology of Medicines, Faculty of Chemistry, Warsaw University of Technology, Warsaw, Poland

**Keywords:** Aquaculture, Synbiotic supplementation, Innate immunity, Environmental friendly

## Abstract

**Background:**

The aim of the study has been to compare the effect of dietary supplementation of β-1,3/1,6-glucan, *Lactobacillus plantarum* bacteria or their mixture on the growth performance, selected parameters of the immune system as well as the liver and intestinal histology of roach. Fish were fed for 14 days with four different diets, each treatment being carried out in triplicate. In control group, fish were fed dry commercial starter feed Aller Performa 2 (Aller Aqua, Denmark). The other experimental fish groups received the same commercial starter feed supplemented with: 1% β-1,3/1,6-glucan (Leiber® Beta-S) in group G; 10^8^ cfu *L. plantarum* g^− 1^ in group L; 1% β-1,3/1,6-glucan + 10^8^ cfu *L. plantarum* g^− 1^ in group G + L. The stimulating effect of the tested preparations was evaluated once the feeding with commercial feed exclusively was resumed and 2 weeks afterwards.

**Results:**

No effect on the survivability and growth performance of the fish was observed in any of the groups. Supplementation of feed with β-1,3/1,6-glucan improved (*P* < 0.05) selected parameters of innate humoral immunity and the pinocytotic activity of phagocytes. Increased respiratory burst activity of head kidney phagocytes (RBA) was observed in groups L and G + L (*P* < 0.05), and the effect persisted for 2 weeks after the commercial feed regime was resumed. An analogous tendency was determined for the killing activity of phagocytes (PKA) of the head kidney with respect to *Aeromonas hydrophila*, although this effect appeared only during the feed supplementation period. Supplying roach with β-1,3/1,6-glucan, singly or with *L. plantarum*, had no effect (*P* > 0.05) on the proliferation of mitogen-activated lymphocytes. However, an increase in the number of CD3-positive cells and goblet cells was noticed in the digestive system of the L group fish (*P* < 0.05).

**Conclusions:**

The results show that feeding fish with added *L. plantarum* and β-1,3/1,6-glucan stimulates the non-specific resistance mechanisms and raises the counts of intestinal immune cells. Synbiotic may help to control serious bacterial diseases and offer an alternative to antibiotics commonly used in fish farming, and its prolonged immunostimulatory effect could increase fish surviving after release to the natural environment.

## Background

In 2018, cyprinid fish constituted most of the fish produced for human consumption in global aquaculture [9]. Consistent efforts to intensify the production of these fish entail a greater risk of diseases. Thus, it becomes crucial to identify possibilities of attaining the improvement of the fish’s health status via treatment with immunostimulating methods which are not harmful to the environment. Equally important is the preparation of fish stocking material distinguished by high survivability rate, which basically depends on the efficiently functioning immune system of fish. Fish released to open waters are exposed to handling and environmental stresses, which is why they need to be in good condition and demonstrate high resistance. Attempts are undertaken to enhance the immunity and to improve the health of fish via natural immunostimulating methods, for instance using efficacious probiotics, prebiotics and synbiotics [24].

The literature data concerning the effective use of synbiotics in aquaculture are rather scarce, but the available results indicate that their administration can be an alternative prophylactic and therapeutic measure. On the one hand, synbiotics inhibit the multiplication of pathogens; on the other hand, they stimulate the defense mechanisms of the host organism by improving its resistance, especially to bacterial infections. They often have a beneficial impact on fish rearing effects. However, the achievement of a good therapeutic outcome depends on an appropriate formulation of a synbiotic and its dosing [4, 8, 10, 23, 41].

There are few preparations available on the market exclusively dedicated to fish farming and adapted to the specific composition of intestinal microflora that will show multi-directional effects and, as such, might be an alternative to antibiotic therapy in aquaculture. The synbiotic preparation which is being designed in our research project is composed of five selected strains of the bacterium *Lactobacillus plantarum*, so as to ensure a broad spectrum of antimicrobial activity. Previous studies conducted on these strains confirmed their antibacterial, autoaggregation properties as well as the ability to survive in the digestive tract of fish [17]. These five strains of lactic acid bacteria were combined with highly purified molecules of β-1,3/1,6-glucan derived from the cell wall of baker’s yeast *Saccharomyces cerevisiae*, which were intended to serve as a substrate for the fermentation carried out by *L. plantarum*, to stimulate the growth and activity of the bacteria, and thereby to facilitate their intestinal colonization and prolong the duration of their influence on the fish body. The remaining amounts of glucan, not metabolized by *L. plantarum*, was expected to improve the fish’s immunity. The preliminary experiment we conducted indicated that a 1% supplement of β-1,3/1,6-glucan to the probiotic-supplemented fish feed was most effective in making an impact on the mechanisms of the immune system.

The chosen therapeutic strategy was based on the concept of creating a synbiotic preparation demonstrating a multi-directional action, used as a dietary supplement, in which both components (probiotic + prebiotic) would interact with each other and improve the immunological parameters as well as the condition of fish. The expected result should be maintained for at least 2 weeks after the discontinuation of supplementation.

The studies on the use of synbiotics in aquaculture focus mainly on cultured species produced for human consumption. In contrast, this study was undertaken to enhance the immunity of the fish reared to restock open waters. Fish released to the natural environment are exposed for the first contact with many of pathogenic agents, that not occur or are rare under the culture conditions. The study was conducted on roach (*Rutilus rutilus*), member of the cyprinid family, which is a popular fish species in Europe. It was assumed for the purpose of this study that this species could serve as a model for cyprinid stock material bred under controlled conditions.

The objective of the research has been to compare the effect of supplementation of a commercial feed with the selected probiotic (*L. plantarum*), prebiotic (β-1,3/1,6-glucan) and a synbiotic produced from these two components, on selected parameters of the roach’s immune system and digestive tract. In addition, the stimulating effect of the selected preparations was assessed 2 weeks after the fish resumed being fed the commercial feed alone.

## Results

### Fish survival and growth performance

No mortality occurred during the experiment. Fish mean specific growth rate (SGR) ranged within 1.37–1.46, and the mean increment in total length (ITL) was 0.26–0.31 mm d^− 1^ at the end of phase 1 of the experiment (Table 1). During phase 2, the fish growth was considerably slower (SGR 0.48–0.69, ITL 0.12–0.16 mm d^− 1^). All differences between mean values of growth parameters were insignificant (*P* > 0.05) at the end of both phases of the experiment.

**Table 1:** Final mean (± SD) values of BW, TL, SGR and daily ITL of *R. rutilus* juveniles fed commercial diet (Control group) and the diet supplemented with: β-glucan (G), *L. plantarum* (L) and β-glucan+*L. plantarum* (G + L)

Experimental period	Parameter	Group			
		Control	G	L	G + L
Phase 1 (experimental days 1–14)	BW (g)	3.00 ± 0.17	3.02 ± 0.29	2.94 ± 0.18	2.95 ± 0.10
	TL (mm)	71.0 ± 1.4	71.0 ± 2.0	70.9 ± 1.1	70.7 ± 0.9
	SGR	1.46 ± 0.08	1.46 ± 0.03	1.41 ± 0.06	1.37 ± 0.12
	ITL (mm d^− 1^)	0.31 ± 0.05	0.26 ± 0.02	0.27 ± 0.04	0.27 ± 0.03
Phase 2 (experimental days 15–28)	BW (g)	3.28 ± 0.35	3.30 ± 0.62	3.24 ± 0.23	3.48 ± 0.20
	TL (mm)	73.3 ± 2.5	73.7 ± 3.7	73.1 ± 1.4	73.9 ± 1.4
	SGR	0.57 ± 0.06	0.48 ± 0.07	0.69 ± 0.16	0.63 ± 0.23
	ITL (mm d^− 1^)	0.16 ± 0.03	0.15 ± 0.02	0.16 ± 0.02	0.12 ± 0.06

The differences between experimental groups are not significant (*n* = 3; *p* < 0.05)

### Evaluation of non-specific humoral immunity and biochemical parameters

The lysozyme (LZ) activity and total immunoglobulin (Ig) level in serum were significantly highest (*P* < 0.05) in G and G + L groups as compared to the Control group and L group both at the end of phase 1 (uptake 1) and end of phase 2 (uptake 2) of the experiment (Tables 2 and 3). The ceruloplasmin (Cp) activity and total protein (TP) level in serum were not significantly different between all the experimental groups (*P* > 0.05).

**Table 2:** The humoral-mediated immune parameters in roach fed β-glucan (G), *L. plantarum* (L) and β-glucan+*L. plantarum* (G + L) supplemented feed or commercial diet (Control), uptake 1

Parameter	Control	G	L	G + L
LZ activity in serum (mg L^− 1^)	36.3 ± 0.3^b^	47.7 ± 0.2^a^	36.5 ± 0.2^b^	49.2 ± 0.3^a^
Cp activity in serum (IU)	46.23 ± 1.94^a^	48.37 ± 1.44^a^	46.58 ± 1.27^a^	47.52 ± 1.84^a^
TP level in serum (g L^− 1^)	25.59 ± 1.84^a^	27.53 ± 1.72^a^	26.77 ± 1.16^a^	28.13 ± 1.04^a^
Total Ig level in serum (g L^− 1^)	5.13 ± 1.68^b^	9.12 ± 1.26^a^	5.82 ± 1.38^b^	9.95 ± 1.79^a^

mean ± SD; *n* = 3; a,b - significant differences between marked values at *p* < 0.05

**Table 3:** The humoral-mediated immune parameters in roach fed β-glucan (G), *L. plantarum* and β-glucan+*L. plantarum* (G + L) supplemented feed or commercial diet (Control), uptake 2

Parameter	Control	G	L	G + L
LZ activity in serum (mg L^−1^)	36.2 ± 0.3^b^	47.8 ± 0.4^a^	36.4 ± 0.2^b^	48.7 ± 0.4^a^
Cp activity in serum (IU)	47.35 ± 0.83^a^	46.98 ± 1.74^a^	47.36 ± 0.56^a^	47.88 ± 1.31^a^
TP level in serum (g L^− 1^)	26.14 ± 2.04^a^	28.29 ± 0.83^a^	27.63 ± 0.86^a^	27.97 ± 1.07^a^
Total Ig level in serum (g L^− 1^)	6.08 ± 1.27^b^	10.12 ± 1.78^a^	7.95 ± 0.83^b^	9.85 ± 1.95^a^

mean ± SD; n = 3; a,b - significant differences between marked values at p < 0.05

### Evaluation of non-specific cellular immunity

The pinocytotic activity of the head kidney phagocytes was significantly highest (*P* < 0.05) in G group at the end of both phase 1 and phase 2 of the experiment (Tables 4 and 5).

**Table 4:** The cell-mediated immune parameters in roach fed β-glucan (G), *L. plantarum* (L) and β-glucan+*L. plantarum* (G + L) supplemented feed or commercial diet (Control), uptake 1

Parameter	Control	G	L	G + L
Pinocytic activity of the head kidney phagocytes (% of ingested NR)	19.84 ± 3.14^b^	29.09 ± 4.84^a^	21.79 ± 4.21^b^	20.76 ± 1.73^b^
RBA of spleen phagocytes (SI)	1.05 ± 0.12^a^	0.97 ± 0.10^a^	1.10 ± 0.15^a^	1.00 ± 0.13^a^
RBA of head kidney phagocytes (SI)	1.15 ± 0.10^b^	1.46 ± 0.14^ab^	1.73 ± 0.20^a^	1.73 ± 0.19^a^
PKA of spleen phagocytes (SI)	1.13 ± 0.11^b^	1.11 ± 0.12^b^	1.44 ± 0.13^a^	1.10 ± 0.06^b^
PKA of head kidney phagocytes (SI)	1.07 ± 0.09^c^	1.13 ± 0.09^c^	1.48 ± 0.09^a^	1.29 ± 0.07^b^
Proliferative response of head kidney lymphocytes stimulated by ConA (SI)	1.47 ± 0.09^a^	1.19 ± 0.12^a^	1.29 ± 0.15^a^	1.21 ± 0.10^a^
Proliferative response of spleen lymphocytes stimulated by ConA (SI)	1.25 ± 0.14^a^	1.32 ± 0.11^a^	1.37 ± 0.17^a^	1.14 ± 0.09^a^
Proliferative response of head kidney lymphocytes stimulated by LPS (SI)	1.26 ± 0.14^a^	1.09 ± 0.10^a^	1.11 ± 0.13^a^	1.05 ± 0.08^a^
Proliferative response of spleen lymphocytes stimulated by LPS (SI)	1.12 ± 0.10^a^	1.09 ± 0.11^a^	1.02 ± 0.09^a^	1.10 ± 0.10^a^

mean ± SD; *n* = 3; a,b,c - significant differences between marked values at *p* < 0.05

**Table 5:** The cell-mediated immune parameters in roach fed β-glucan (G), *L. plantarum* and β-glucan+*L. plantarum* (G + L) supplemented feed or commercial diet (Control), uptake 2

Parameter	Control	G	L	G + L
Pinocytic activity of the head kidney phagocytes(% of ingested NR)	17.50 ± 2.08^b^	23.90 ± 1.54^a^	17.20 ± 3.24^b^	18.74 ± 1.65^b^
RBA of spleen phagocytes (SI)	1.15 ± 0.14^a^	1.21 ± 0.21^a^	1.29 ± 0.08^a^	1.05 ± 0.09^a^
RBA of head kidney phagocytes (SI)	2.10 ± 0.43^a^	2.03 ± 0.15^a^	2.36 ± 0.22^a^	2.17 ± 0.30^a^
PKA of spleen phagocytes (SI)	1.39 ± 0.10^a^	1.48 ± 0.25^a^	1.29 ± 0.13^a^	1.35 ± 0.10^a^
PKA of head kidney phagocytes (SI)	1.38 ± 0.10^c^	1.56 ± 0.07^c^	1.65 ± 0.09^a^	1.61 ± 0.11^b^
Proliferative response of head kidney lymphocytes stimulated by ConA (SI)	1.28 ± 0.10^a^	1.49 ± 0.16^a^	1.37 ± 0.14^a^	1.33 ± 0.12^a^
Proliferative response of spleen lymphocytes stimulated by ConA (SI)	1.15 ± 0.12^a^	1.08 ± 0.11^a^	1.29 ± 0.10^a^	1.28 ± 0.15^a^
Proliferative response of head kidney lymphocytes stimulated by LPS (SI)	1.11 ± 0.11^a^	1.24 ± 0.09^a^	1.21 ± 0.13^a^	1.14 ± 0.11^a^
Proliferative response of spleen lymphocytes stimulated by LPS (SI)	1.07 ± 0.09^a^	1.05 ± 0.11^a^	1.20 ± 0.08^a^	1.18 ± 0.12^a^

mean ± SD; n = 3; a,b,c - significant differences between marked values at *p* < 0.05

The respiratory burst activity (RBA) of the head kidney phagocytes was significantly highest (*P* < 0.05) in L and G + L groups at the end of phase 1 only (Tables 4 and 5). No significant differences were observed between the results of the RBA of spleen phagocytes in all groups at the end of phase 2. The potential killing activity (PKA) of head kidney phagocytes was significantly highest (*P* < 0.05) in L and G + L groups at the end of both experimental phases (Tables 4 and 5). PKA of spleen phagocytes was significantly highest (*P* < 0.05) in L group, but only at the end of phase 1 of the experiment (Tables 4 and 5).

### Proliferative response of lymphocytes – MTT reduction assay

The proliferative response of the spleen and head kidney lymphocytes stimulated by lipopolysaccharide (LPS) and concanavalin A (ConA) was not significantly different between the G, L, G + L and Control groups (*P* > 0.05) (Tables 4 and 5).

### Histology of digestive tract

There were no abnormalities in the structure of the gastrointestinal tract, including the absence of histopathological changes, in any of the examined groups. The longest intestinal folds were observed in the anterior and midgut in the Control group, and in the posterior intestine in L group (Table 6). Numerous periodic acid-Schiff (PAS) positive protein absorption granules were found in the posterior intestine in the supranuclear space of enterocytes in all the examined groups.

**Table 6:** Histomorphometric results of the intestine and liver of *R. rutilus* juveniles fed commercial diet (Control) and the diet supplemented with: β-glucan (G), *L. plantarum* (L) and β-glucan+*L. plantarum* (G + L) (±SD)

Parameter	Control	G	L	G + L
Anterior fold length [μm]	342.23 ± 39.17^a^	282.47 ± 93.87^b^	313.79 ± 40.73^ab^	317.54 ± 32.61^ab^
Anterior CD3-positive cells/100 μm fold length	27.19 ± 5.07^b^	40.05 ± 11.97^a^	25.93 ± 3.27^b^	35.35 ± 4.33^a^
Anterior PCNA-positive/100 μm fold length	8.91 ± 1.68^b^	18.02 ± 2.32^a^	26.67 ± 4.94^c^	27.93 ± 5.06^c^
Anterior rodlet cells/100 μm fold length	0.65 ± 0.41^b^	2.13 ± 0.57^c^	2.64 ± 0.53^c^	8.73 ± 1.07^a^
Midgut fold length [μm]	292.48 ± 32.78^b^	249.34 ± 51.27^b^	274.69 ± 32.31^b^	210.71 ± 23.95^a^
Midgut CD3-positive cells/100 μm fold length	14.89 ± 3.83^b^	17.22 ± 3.30^b^	15.49 ± 2.64^b^	35.82 ± 4.72^a^
Midgut PCNA-positive cell/100 μm fold length	4.71 ± 0.99^b^	9.48 ± 2.32^c^	11.14 ± 1.29^c^	26.34 ± 4.19^a^
Midgut rodlet cells/100 μm fold length	1.96 ± 0.57^c^	1.91 ± 0.58^c^	3.38 ± 0.92^b^	7.31 ± 2.05^a^
Posterior fold length [μm]	290.29 ± 54.14^b^	297.76 ± 53.93^b^	330.11 ± 59.35^b^	248.32 ± 29.04^a^
Posterior CD3-positive cells/100 μm fold length	44.11 ± 9.46^ab^	48.94 ± 12.29^a^	34.22 ± 7.70^c^	39.36 ± 3.62^bc^
Posterior PCNA-positive cells/100 μm fold length	12.78 ± 1.61^b^	16.53 ± 3.59^a^	11.63 ± 1.73^b^	18.87 ± 4.23^a^
Number of PCNA-positive cells in liver/100 μm^2^	0.006 ± 0.003^c^	0.016 ± 0.004^bc^	0.011 ± 0.008^b^	0.031 ± 0.007^a^

^a,b,c^ - significant differences between marked values at p < 0.05

**Fig. 1:**
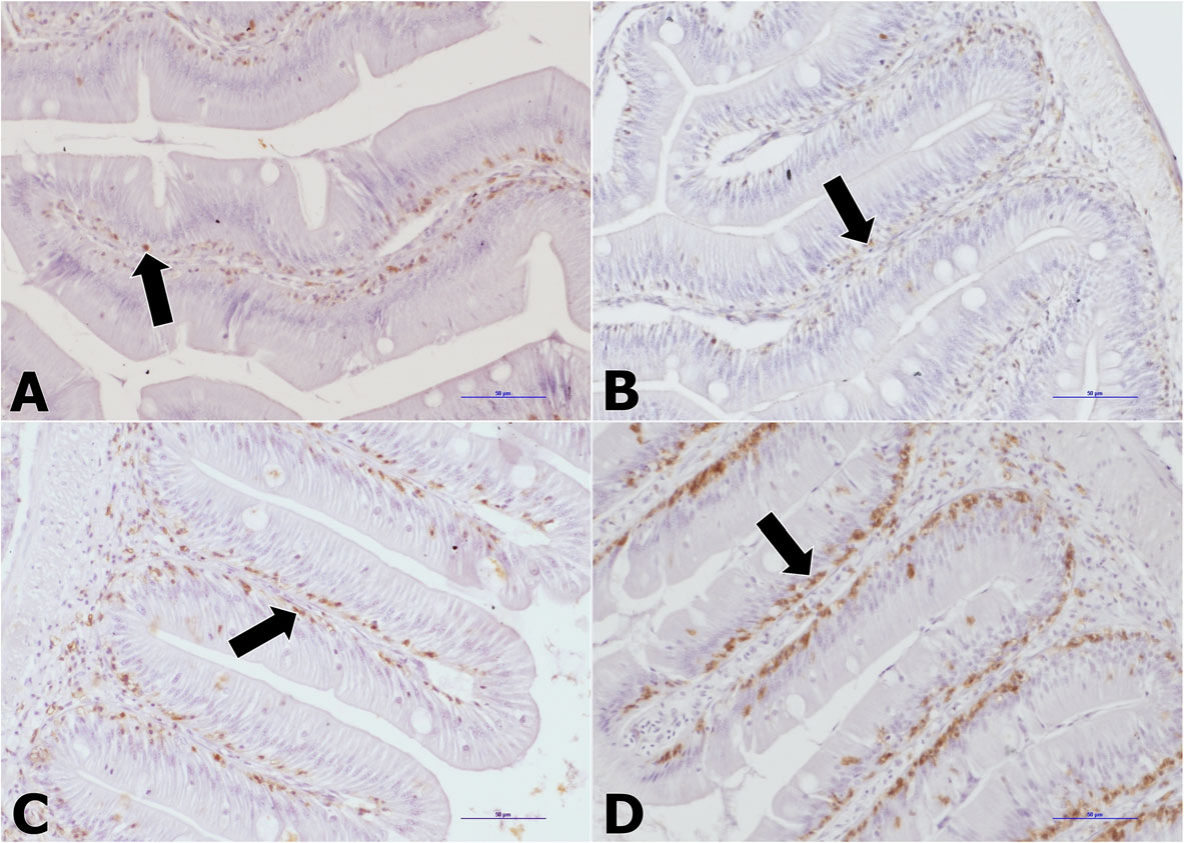
Immunohistochemical localization of CD3-positive cells (black arrows) in the midgut of *Rutilus rutilus* juveniles fed commercial diet: A - Control group and the diet supplemented with: B - β-glucan (G group), C - *L. plantarum* (L group) and D - β-glucan+*L. plantarum* (G + L group), mag. 200x

The highest number of PCNA-positive nuclei was observed in intestinal folds in all investigated parts of the digestive tract in G + L group. The CD3-positive cells occurred in the distal part of the intestine fold, under enterocytes (Fig. 1). In the anterior intestine, the highest number of CD3-positive cells was found in G and G + L groups, while in the posterior intestine they were most numerous in G group (Table 6).

Rodlet cells were present in the anterior and middle intestine, located between enterocytes and mucous cells. Rodlet cells were characterized by a distal cell nucleus, current PAS positive-granules in the supranuclear cytoplasm, and a calyx shape. It was impossible to determine the number of rodlet cells in the posterior segment of the intestine in any of the examined groups due to the numerous granules of absorbed protein. In the anterior and middle part of the intestine, the number of rodlet cells was significantly highest in G + L group.

There were no histopathological changes in the liver and pancreas, same as in the intestines. The highest number of PCNA-positive cells in the liver was found in G + L group. Hepatocytes were characterized by clearly visible fat drops in all groups. The liver had the highest average number of proliferating cells in G + L group and the lowest in the Control group (Table 6).

## Discussion

The evidently growing market demand for pro-health preparations has encouraged us to conduct this study, where effects of a probiotic composed of selected strains of the bacterium *L. plantarum*, a prebiotic such as β-1,3/1,6-glucan, and a synbiotic formulated by mixing these two substances on parameters of the immune system and growth performance of roach were investigated.

No significant influence of the examined synbiotic on the rearing parameters of roach were determined, which agrees with results of previous studies on European seabass (*Dicentrarchus labrax*), Nile tilapia (*Oreochromis niloticus*), and channel catfish (*Ictalurus punctatus*) [3, 39, 40]. Although synbiotics are thought to be growth stimulators, and their beneficial effect on fish rearing parameters, growth in particular, has been observed, it is possible that the duration of their application tested in our study on roach was insufficient [6]. Synbiotics act on the digestive tract, increasing the efficiency of the intestinal absorbtion surface owing to the presence of probiotic bacteria, which support the natural process of hydrolysis of proteins to peptides and amino acids [19]; subsequently some of these products participate in the synthesis of short-chain fatty acids (SCFAs), which stimulate the proliferation and permeability of intestines, thereby increasing the absorbability of nutrients [13].

The experiment findings suggest that addition of β-1,3/1,6-glucan can cause increase in the levels of LZ and total Ig, both of which belong to the non-specific humoral immune response and protect an organism during a time period when it has not yet developed the acquired immune response mechanisms. Similar results were achieved by Ye et al. (2011) [41], who studied Japanese flounder (*Paralichthys olivaceus*) fed a diet supplemented with synbiotics. Higher levels of LZ and total Ig were also observed after diets given to different fish species had been enriched solely with β-glucan [1, 21, 24, 28, 34]. The results of Cp activity suggested that β-1,3/1,6-glucan and *L. plantarum* had no negative influence on hepatocytes, nor did they implicate the presence of acute phase proteins, which are a heterogeneous group of serum proteins synthesized in the liver [34].

The effects of our experiment suggest that the increase in the metabolic activity of phagocytes was associated with the supply of *L. plantarum* bacteria. RBA is one of the principal parameters of innate immune response and is broadly used as an indicator of the organism’s immunological activity. Probiotics can stimulate phagocytes to produce more reactive oxygen species, which are toxic to bacteria, fungi and parasites [27]. There are reports indicating that the supplementation of fish diets with lactic acid bacteria (LAB) has a considerable impact on RBA. Similar results were observed in studies on roho labeo (*Labeo rohita*), grouper (*Epinephelus coioides*), common carp (*Cyprinus carpio*), rainbow trout (*Oncorhynchus mykiss*), basa catfish (*Pangiasius bocourti*), Nile tilapia (*Oreochromis niloticus*) [11, 12, 18, 20, 26, 29, 30, 35, 37, 42]. In turn, Ai et al. (2007) noticed that the supplementation of β-glucan did not raise the phagocytic activity of the head kidney macrophages in large yellow croaker (*Pseudosciaena crocea*) [1]. Likewise, Akrami et al. (2013) did not observe changes in RBA in stellate sturgeon juvenile (*Acipenser stellatus*) after dietary supplementation with FOS [2]. All the cited papers support the results obtained in the present study.

An analogous tendency was observed in the PKA of phagocytes. The results show that the increase in PKA was independent of β-1,3/1,6-glucan; instead, it was a response to the influence of *L. plantarum*. An increase in PKA points to a higher degree of antibacterial protection in fish which receive in their diet an addition of the tested strains of bacteria, an effect that persisted for two more weeks after the fish resumed to be fed the unsupplemented commercial feed. The literature provides evidence supporting similar tendencies in many fish species, e.g. cobia (*Rachycentron canadum*), grouper, rainbow trout and common carp [10, 18, 25, 30, 35].

The elevated pinocytotic activity (ability to absorbe micromolecular substances) in fish fed commercial feed supplemented with β-1,3/1,6-glucan, remains in concordance with results obtained in rainbow trout by Verlhac et al. (1998) [38]. The pinocytosis assay was shown to be a biomarker for the immune response of fish [5].

Supplementation of diets with β-1,3/1,6-glucan and with *L. plantarum* did not influence the proliferative activity of mitogen-stimulated lymphocytes, although our previous experiments showed a significant increase in the proliferative activity of B lymphocytes stimulated by bacterial LPS in carp fingerlings which received a feed supplemented with the same strains of *L. plantarum* [18]. In this case, the result may have been due to the insufficient time for optimal induction of specific immune response [14]. However, the number of immune cells, rodlet cells and proliferating cells detected in the intestines of fish from synbiotic-supplemented group indicates its localized (direct) beneficial influence on the intestinal tract. Other research reports indicate that dietary Macrogard in tench (*Tinca tinca*) and β-glucan Leiber®Beta-S supplied to European eel (*Anguilla anguilla*) stimulated the activity of T and B lymphocytes [32, 34]. With respect to tench, higher counts of T lymphocytes, which are a fraction of IEL (intra epithelial leucocytes), and goblet cells in the intestinal tract of fish administered probiotic bacteria might indicate the stimulating effect of the supplement. Similar findings have been reported in studies on other fish species, for example Nile tilapia [36].

Stimulation of the innate cellular and humoral immunity plays a significant role in the prevention of infectious diseases of fish, especially ones caused by facultative pathogenic microorganisms. Supplementation of a diet with β-1,3/1,6-glucan demonstrated a stimulating effect on the non-specific humoral immunity of roach, leading to higher levels of LZ and total Ig, which perform important antibacterial roles. In turn, the supplementation of fish diets with *L. plantarum* resulted in the increase of the activity of phagocytes, which is a very important development as these cells participate in the engulfing and killing of microorganisms, in addition to which they co-act with T lymphocytes and play a role in the antibacterial response.

## Conclusions

The results suggest that the dietary supplementation ingredients used in the experiment interacted with each other, and although the tested synbiotic formulation did not fully attain the expected outcomes, one cannot exclude that this synbiotic can be used in aquaculture successfully to stimulate the non-specific immunity of fish. In our opinion, it is worth continuing studies on the other fish species diet supplemented with probiotic strains of *L. plantarum* and with β-1,3/1,6-glucan in order to develop an optimal application technology. Such mixture of the components may help to control serious bacterial diseases and offer an alternative to antibiotics and chemotherapeutic agents commonly used in fish farming. In addition, the potential long-time effect may increase fish surviving after release to the natural environment thus being an important factor in open waters restocking. An opportunity to apply feeds enriched with natural additives stimulating the immune system and not affecting adversely the fish rearing results is a compromise between the expectations of fish farmers and of the society.

## Methods

### Experimental fish

Experimental fish originated from own broodstock kept under controlled conditions in Pond Fishery Department, IFI, Zabieniec, Poland. Roach (*R. rutilus*) progeny of three female and three male spawners were pooled. Juveniles began to be prepared for the experiment 8 months after hatching, when their mean wet body weight and total length reached 2.40 ± 0.59 g and 6.66 ± 0.49 cm, respectively.

### Experimental conditions

The healthy fish were stocked into 12 40-l glass flow-through aquaria. Initial stocking density per aquarium was 20 fish. Aquaria were continuously supplied with filtered and aerated water from a recirculating aquaculture system (RAS) at approximately 0.3 l min^− 1^ and heated to 24.9 ± 0.3 °C. Aeration was provided by airstones to maintain the oxygen concentration in the water above 72% of saturation. Other monitored water-quality parameters were determined weekly in one aquarium per group. Total ammonia was 0.31 ± 0.06 mg l^− 1^, nitrites 0.06 ± 0.03 mg l^− 1^, conductivity 402 ± 24 μS cm^− 1^ and pH 7.9 ± 0.3 (mean ± SD). Aquaria were illuminated from 08:00 to 21:00 by fluorescent tubes. Light intensity at the water surface was about 700 lx.

### Experimental design

Fish were randomly divided into four equal groups, in three replicates (*n* = 3): the control group (C) and three experimental groups (G, L and G + L). The study involved two phases.

In phase 1 of the experiment (days 1–14), fish were fed for 14 days with four different diets. In control group, fish were fed dry commercial starter feed Aller Performa 2 (Aller Aqua, Denmark) without any supplementation. Dry feed proximate composition was (mean ± SD): moisture 6.4 ± 0.0%, ash 9.87 ± 0.01, crude protein 54.1 ± 0.3, total lipids 14.1 ± 0.1. Chemical analysis were performed according to the method describe by Kamiński et al. (2017) [16]. The other experimental fish groups were fed the commercial starter feed supplemented with: 1% β-1,3/1,6-glucan (Leiber® Beta-S) (G group); 10^8^ cfu *L. plantarum* g^− 1^ (L group); 1% β-1,3/1,6-glucan + 10^8^ cfu *L. plantarum* g^− 1^ (G + L group)*.* In our study we used a commercial product by Leiber Gmbh named Leiber®Beta-S with a molecular mass of 100–200 kDa. The experiment involved five strains of *L. plantarum* obtained from the collection of strains of the Department of Molecular Biochemistry of the Institute of Biochemistry and Biophysics of the Polish Academy of Sciences in Warsaw, Poland [18]. Prebiotic-supplemented diet (G group) was prepared with β-1,3/1,6-glucan in dosage of 10 g kg^− 1^ feed. The quantity of β-glucan was mixed thoroughly with 3 mL of distilled water and added to 50 g of base feed and then sealed in an AGA Labor vacuum pump (Lublin, Poland). Probiotic-supplemented diet (L group) was prepared according to Kazuń et al. (2018) [18]. The probiotic mixture was mixed thoroughly with 50 g of commercial feed to achieve a dose of ~ 10^8^ cells g^− 1^ of feed. Synbiotic-supplemented diet (L + G group) was prepared by combination of the two above methods. At first there was prepared the feed supplemented with β-glucan, then the probiotic was added. The modified feeds were stored in screw-top glass bottles at room temperature until required. To ensure high probiotic level in the supplemented feed [7, 15], fresh diets were prepared on weekly basis. Initially, the daily food ratio was 1.0 g per aquarium. Feed was given manually at 08:00, 14:00 and 20:00 in equal portions.

In phase 2 of the experiment (days 15–28), all fish were fed non-supplemented commercial feed alone for the next 14 days. Due to the reduction of fish stocks, the daily feed ration was lowered to 0.5 g per aquarium.

### Sample collection

After 14 days of feeding with the experimental diets (phase 1 of the experiment), half the fish were anaesthetized and measurements of their individual BW and TL were performed. From each experimental aquarium, 10 fish were taken randomly, euthanized by immersion with an overdose (50 mg l^− 1^) of unbuffered tricaine methanesulfonate solution (MS-222, Sigma-Aldrich, St. Louis, MO, USA) before sampling. The fish blood, liver, spleen, head kidney and intestinal tract samples were taken (uptake 1). Blood was collected from the caudal vein, and transferred to Eppendorf tubes. Following centrifugation (2000 g, 10 min, 4 °C), serum was collected and stored at − 20 °C until use. At the end of the experiment, all the remaining fish were sacrificed with an overdose of unbuffered tricaine methanesulfonate solution (like at the end of phase 1), individual BW and TL were determined, and then the samples of tissues were taken (uptake 2).

### Evaluation of non-specific humoral immunity parameters

The LZ activity in the plasma was measured in a turbidimetric assay described by Siwicki and Anderson (1993) [31]. The Cp activity in the plasma was determined according to the method developed by Siwicki and Studnicka (1986) [33] and modified for micro-methods. Total Ig levels in serum were also measured using the Lowry micro-method modified by Siwicki and Anderson (1993) [31]. TP levels in serum were determined with the spectrophotometric micro-method proposed by Lowry et al. (1951) [22] and modified by Siwicki and Anderson (1993) [31].

### Isolation of roach immune cells

Roach head kidneys and spleens were pooled within the control and experimental groups (organs from 10 individuals kept in the same aquarium). Organ immune cells were isolated using Histopaque 1077 (Sigma-Aldrich) density gradient centrifugation, suspended at a concentration of 1 × 10^6^ cells ml^− 1^ in RPMI-1640 medium supplemented with 10% fetal calf serum and 1% antibiotic-antimycotic solution (both reagents from Sigma-Aldrich), and cultured/incubated as described before [18]. Isolated cells were then used for assays of pinocytosis, RBA, PKA and proliferative response of lymphocytes (MTT assay). Samples obtained from each pool were tested in duplicate.

### Pinocytosis assay – neutral red uptake (NRU) assay

The pinocytosis assay was performed using a commercially available kit from Sigma-Aldrich (TOX-4), as described earlier [18]. Briefly, after the removal of non-adherent immune cells (lymphocytes), the adherent cells (phagocytes) were incubated in fresh medium containing 0.033% of neutral red for 3 h at 22 °C in order to allow pinocytosis. After cell washing, the solubilisation solution was added to each sample and the absorbance was measured at a wavelength of 540 nm with 690 nm as a reference wavelength using the Sunrise Absorbance Reader (Tecan, Austria). The values were compared to the optical density (OD) of baseline neutral red solution (without cells) and expressed as a percentage of ingested dye.

### RBA and PKA tests

The intracellular RBA and PKA of phagocytes were determined as described before [18]. In short, the adherent immune cells were incubated in fresh medium containing 0.1% NBT (nitroblue tetrazolium, Sigma-Aldrich) and PMA (phorbol myristate acetate, Sigma-Aldrich; 1 μg ml^− 1^) or *A. hydrophila* (1 × 10^8^ cells ml^− 1^) for 60 min at 22 °C. Once the supernatant was removed, cells were fixed with absolute ethanol and the reduced NBT was extracted using KOH and DMSO. The OD of samples was measured colorimetrically at 620 nm. The results were expressed as a stimulation index (SI), which was calculated by dividing the mean OD of PMA (RBA test) or bacteria-stimulated cells (PKA test) by the OD of control, unstimulated cells.

### Proliferative response of lymphocytes – MTT reduction assay

The mitogenic response of roach lymphocytes was determined using the MTT colorimetric assay, as described before [18]. The head kidney or spleen immune cells were cultured in the presence of mitogens – ConA as a T-cell mitogen or LPS from *Escherichia coli* as a B-cell mitogen (both mitogens purchased from Sigma-Aldrich and used in concentrations of 50 μg ml^− 1^) for 72 h at 22 °C. Control, unstimulated cells were maintained in a medium without mitogens. Following incubation, 10 μl of 3-(4,5 dimethylthiazol-2-yl)-2,5-diphenyltetrazolium bromide (MTT, Sigma-Aldrich) solution (10 mg ml^− 1^) were added to each well and the plate was incubated for the next 3 h. After the removal of the supernatant, the reduced MTT was dissolved in DMSO and the optical density was measured at a wavelength of 570 nm with 640 nm as a reference wavelength. The results were expressed as a SI, which was calculated by dividing the mean OD of mitogen-stimulated cells by the OD of control, unstimulated cells.

### Liver and intestinal histology

The intestinal tract and liver samples were fixed with Bouin’s solution and subjected to standard histological procedures. Specimens embedded in paraffin were cut into series of 5 μm thick sections using a Leica (RM2265) microtome (Leica Microsystems, Wetzlar, Germany). Sections were stained by the hematoxylin and eosin (HE) and alcian blue/periodic acid-Schiff (AB/PAS) method and subjected to morphometric analysis (intestine fold height, number of rodlet cells in intestinal epithelium). The immunohistochemical frequency of CD3 and proliferating cells in the intestinal epithelium and liver were assayed on samples stained according to the manufacturer’s protocol of anti-CD3 (Bond™ Ready-to-Use Primary Antibody CD3, LN10, Leica Newcastle, UK) and anti-PCNA (proliferating cell nuclear antigen, clone PC10, DAKO, Poland) antibodies. Colometrical detection of these cells was performed by DAB (3,3′-Diaminobenzidine, Novolink Polymer Detection Kit, Novocastra, Leica, Newcastle, UK). All intestinal measurements were calculated by 100 μm fold length and analysed by One Way Anova with NIR Fisher post hoc test (Statistica 13, Statsoft, Tulsa, OK, USA).

### Calculations and statistical analysis

Individual BW of all fish was determined to the nearest 0.01 g. SGR was calculated from the natural logarithm of the mean final BW minus the natural logarithm of the mean initial BW divided by the total number of experimental days:

$$ \mathrm{SGR}=100\left(\mathrm{Ln}\ {\mathrm{BW}}_{\mathrm{final}}-\mathrm{Ln}\ {\mathrm{BW}}_{\mathrm{initial}}\right)\times {\mathrm{days}}^{-1} $$

Individual fish TL was measured to the nearest 0.1 mm. Daily ITL (mm day^− 1^) was calculated as the mean final TL minus the mean initial TL divided by the total number of experimental days:

$$ \mathrm{ITL}=\left({\mathrm{TL}}_{\mathrm{final}}-{\mathrm{TL}}_{\mathrm{initial}}\right)\times {\mathrm{days}}^{-1} $$

Data were analysed statistically by one-way analysis of variance (ANOVA). The Bonferroni’s *post-hoc* test was used to determine differences between groups (*P* < 0.05). Evaluation of the results was performed with the help of a GraphPadPrism software package.

## Data Availability

The dataset used and/or analysed during the current study are available from the corresponding author on reasonable request.

## Abbreviations

AB/PAS: Alcian blue/periodic acid-Schiff
BW: Wet body weight
ConA: Concanavalin
Cp: Ceruloplasmin
FOS: Fructooligosaccharide
HE: Hematoxylin and eosin
IEL: Intra epithelial leucocytes
ITL: Increment in total length
LAB: Lactic acid bacteria
LPS: Lipopolysaccharide
LZ: Lysozyme
OD: Optical density
PAS: Periodic acid-Schiff
PKA: Potential killing activity
RAS: Recirculating aquaculture system
RBA: Respiratory burst activity
SCFAs: Short-chain fatty acids
SGR: Specific growth rate
SI: Stimulation index
TL: Total length
Ig: Immunoglobulin
TP: Total protein
